# Inhaled corticosteroids have a protective effect against lung cancer in female patients with chronic obstructive pulmonary disease: a nationwide population-based cohort study

**DOI:** 10.18632/oncotarget.15386

**Published:** 2017-02-16

**Authors:** Shih-Feng Liu, Ho-Chang Kuo, Meng-Chih Lin, Shu-Chen Ho, Mei-Lien Tu, Yu-Mu Chen, Yung-Che Chen, Wen-Feng Fang, Chin-Chou Wang, Guan-Heng Liu

**Affiliations:** ^1^ Division of Pulmonary and Critical Care Medicine, Department of Internal Medicine, Kaohsiung, Taiwan; ^2^ Department of Respiratory Therapy, Kaohsiung Chang Gung Memorial Hospital, Kaohsiung, Taiwan; ^3^ Chang Gung University College of Medicine, Kaohsiung, Taiwan; ^4^ Department of Pediatrics, Kaohsiung Chang Gung Memorial Hospital, Kaohsiung, Taiwan; ^5^ Department of Senior High School, Li-Chih Valuable School, Kaohsiung, Taiwan

**Keywords:** inhaled corticosteroids, chronic obstructive pulmonary disease, lung cancer, incidence, hazard ratio

## Abstract

Whether the use of inhaled corticosteroids (ICS) protects patients with chronic obstructive pulmonary disease (COPD) from lung cancer remains undetermined. In this retrospective nationwide population-based cohort study, we extracted data of 13,686 female COPD patients (ICS users, *n =* 1,290, ICS non-users, *n =* 12,396) diagnosed between 1997 and 2009 from the Taiwan's National Health Insurance database. These patients were followed-up until 2011, and lung cancer incidence was determined. Cox regression analysis was used to estimate hazard ratios (HRs) for lung cancer incidence. The time to lung cancer diagnosis was significantly different between ICS users and non-users (10.75 vs. 9.68 years, *P* < 0.001). Per 100,000 person-years, the lung cancer incidence rate was 235.92 for non-users and 158.67 for users [HR = 0.70 (95% confidence interval {CI}: 0.46–1.09)]. After adjusting for patients' age, income, and comorbidities, a cumulative ICS dose > 39.48 mg was significantly associated with a lower risk of lung cancer [ICS users > 39.48 mg, HR = 0.45 (95% CI: 0.21–0.96)]. Age ≥ 60 years, pneumonia, diabetes mellitus, and hypertension decreased lung cancer risk, whereas pulmonary tuberculosis increased the risk. Our results suggest that ICS have a potential role in lung cancer prevention among female COPD patients.

## INTRODUCTION

Chronic obstructive pulmonary disease (COPD) is characterized by persistent airflow limitation that is usually progressive and associated with an enhanced chronic inflammatory response in the airways and lungs to noxious particles or gases [1]. Exposure to cigarette smoke and other noxious particles is an important risk factor for COPD. Smoking is also a risk factor for lung cancer. COPD patients have a high incidence of smoking and higher incidence to develop lung cancer than non-COPD smokers [2, 3]. The risk of lung cancer in COPD patients owing to the COPD itself vs. smoking has been investigated in the past. The evidence suggests that COPD is an independent risk factor for lung cancer [4–6]. Studies have shown that patients with mild or moderate/severe obstructive pulmonary disease have a significantly higher incidence of lung cancer than non-COPD patients after adjusting for factors related to smoking [4], and similar results have been reported in other studies after matching or adjusting for age, sex, and smoking status [5, 6]. Additionally, there is a positive relationship between COPD severity and the development of lung cancer [7].

The Global Initiative for Chronic Obstructive Pulmonary Disease (GOLD) recommends that patients with mild to moderate COPD be predominantly treated with inhaled bronchodilators. Inhaled corticosteroids (ICS) are suggested as a treatment for patients with severe and very severe COPD, especially those with repeated exacerbations [1] and a post-bronchodilator predicted forced expiratory volume in the first second (FEV1) < 50%, with one or more acute exacerbations per year or more than one hospitalization per year [8]. Regular ICS treatment improves symptoms and quality of life, and decreases the frequency of exacerbations [9].

Chronic inflammation may be a causative factor in a variety of cancers. Generally, the longer the inflammation lasts, the higher the risk of cancer. Long-term exposure to inflammatory mediators results in increased cell proliferation, mutagenesis, oncogene activation, and angiogenesis. The end result is a loss of normal growth-controlled cell proliferation [10, 11]. The mechanisms by which COPD patients develop lung cancer are not well established. There is growing evidence that the two diseases have common causes, such as the same underlying tendency, underlying genetic predisposition, telomere shortening, mitochondrial dysfunction, or premature aging [12]. COPD may be a driving factor in lung cancer as it can increase oxidative stress and the result in DNA damage, thereby increasing long-term exposure to proinflammatory cytokines, inhibiting DNA repair mechanisms, and leading to increased cell proliferation [12]. Moreover, lung cancer develops in a host environment in which the dysregulated inflammatory response may promote tumor progression. Chronic inflammation in COPD via the aforementioned mechanisms may drive lung cancer development; thus, the use of anti-inflammatory drugs as anti-cancer treatment is gaining interest in the field. Preclinical studies have also demonstrated that glucocorticoids inhibit the growth of lung cancer cells [13, 14]. ICS have been shown to regulate the production of prostaglandin E2 via cyclooxygenase-2 (COX-2) [15], as well as inhibiting proto-oncogenes in smokers [16]. In addition, ICS have been shown to reduce local and systemic inflammation among patients with COPD [17–19]. Another prospective observational study demonstrated that a high ICS dose is correlated with a decreased risk of lung cancer [20]. However, there are many conflicting ideas in the literature about the role of ICS in the prevention of lung cancer [20–27]. Taiwan's National Health Insurance (NHI) database may provide a larger population and longer follow-up interval data than previous studies. Therefore, we conducted a nationwide population-based cohort study to investigate whether the use of ICS reduces the incidence of lung cancer.

## RESULTS

### Patient characteristics

**Figure 1:**
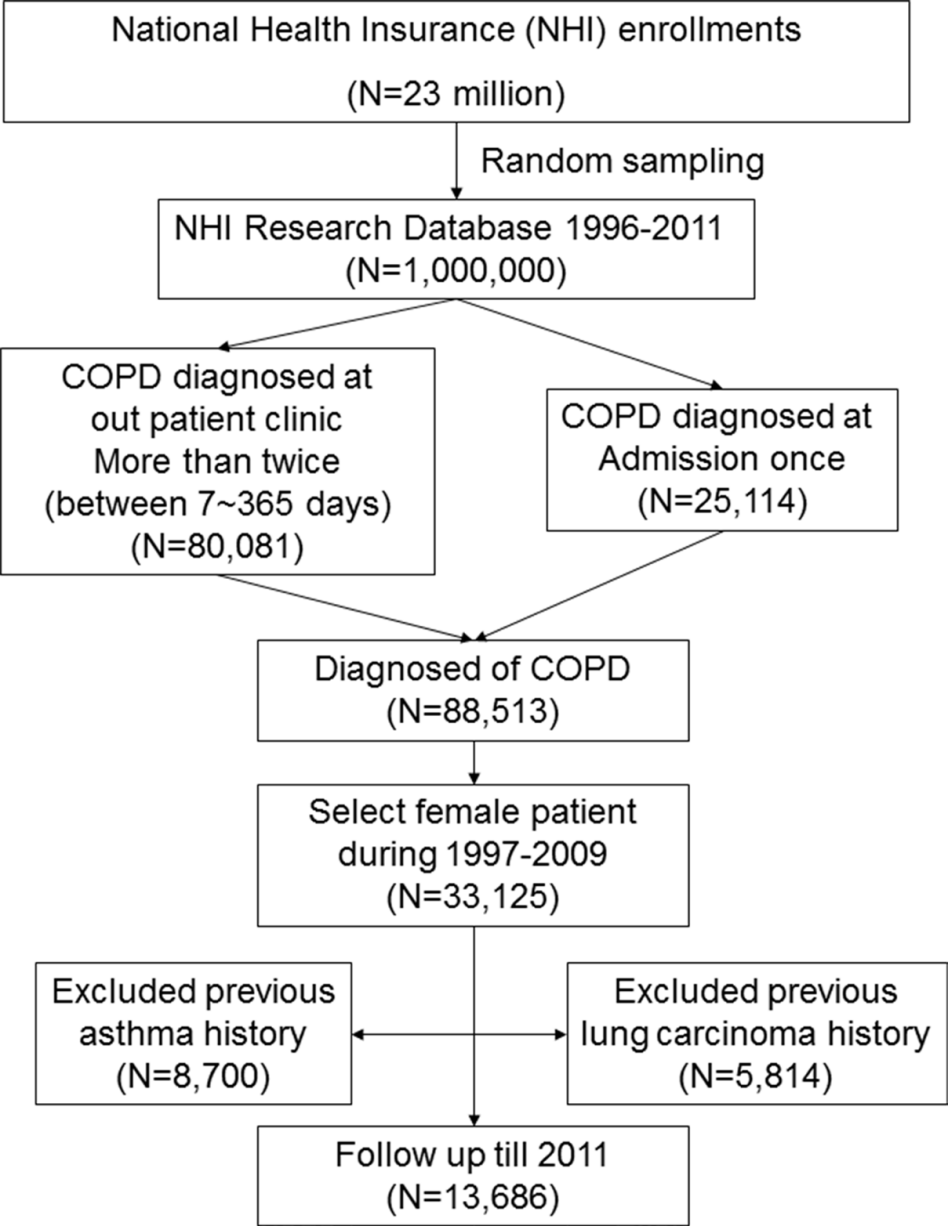
Flow chart for patient selection Note : This is a retrospective, population-based study in which we extracted data of female patients newly diagnosed with COPD between 1997 and 2009 from the Taiwan's National Health Insurance database (ICD-9-CM 491, 492, 496). Patients with COPD were defined by the presence of two or more diagnostic codes for COPD within 12 months. Patients were excluded if they were < 40 years, if lung cancer had been diagnosed prior to the diagnosis of COPD, or if the patient had cases of asthma (ICD-9 CM code 493.X) before the index date. The enrolled patients (*n* = 13,686) were followed-up until 2011, and the incidence of lung cancer was determined. Abbreviations: COPD: chronic obstructive pulmonary disease; ICD-9-CM, International Classification of Diseases, Ninth Revision–Clinical Modification.

We enrolled 13,686 female patients with COPD in the present study (Figure 1). Demographic characteristics of the patients are listed in Table 1. Among this population, 8,977 patients (65.59%) were older than 60 years, and 940 of these patients had been administered ICS; 7,953 patients (58.11%) had an income of less than 15,847 New Taiwan Dollar (NTD) per month, and 941 of these patients had been administered ICS; and 10,226 patients (74.72%) had hypertension, and 1,203 of these patients had been administered ICS. Oral steroid use was prescribed in 1,683 patients (12.3%). Of 1,290 ICS users, 285 (22.09%) had ever been prescribed an oral steroid. Oral steroid use was significantly different between ICS users and non-users (22.09% vs. 11.28%, *P <* 0.001).

**Table 1:** Demographic characteristics of patients in the cohort

Variables	All patients		Patient with ICS use	Without ICS use	*p*-value
	*n*	%	*n* (%)	*n* (%)	
Follow_up (years)	9.78 ± 3.32		10.75 ± 2.91	9.68 ± 3.35	< .0001
Age					
≥ 40~< 50	2077	15.18	178 (13.80)	1899 (15.32)	0.0672
≥ 50~< 60	2632	19.23	268 (20.78)	2364 (19.07)	
≥ 60	8977	65.59	844 (65.43)	8133 (65.61)	
Income (NTD)					
< 15840	7953	58.11	794 (61.55)	7159 (57.75)	0.1811
≥ 15840~< 21900	1659	12.12	141 (10.93)	1518 (12.25)	
≥ 21900~< 34800	3169	23.16	273 (21.16)	2896 (23.36)	
≥ 34800	905	6.61	82 (6.36)	823 (6.64)	
Oral steroid					
No use	12003	87.70	1005 (77.91)	10998 (88.72)	< .0001
Any use	1683	12.30	285 (22.09)	1398 (11.28)	
Medical disease					
Pulmonary tuberculosis	983	7.18	158 (12.25)	825 (6.66)	< .0001
Bacterial pneumonia	1161	8.48	225 (17.44)	936 (7.55)	< .0001
Bronchiectasis	1270	9.28	259 (20.08)	1011 (8.16)	< .0001
Pulmonary Fibrosis	155	1.13	44 (3.41)	111 (0.90)	< .0001
Hypertension	10226	74.72	1038 (80.47)	9188 (74.12)	< .0001
Diabetes mellitus	6062	44.29	683 (52.95)	683 (43.39)	< .0001

Abbreviations: NTD: New Taiwan Dollar.

### Protective effects of ICS on lung cancer in patients with chronic obstructive pulmonary disease

**Figure 2:**
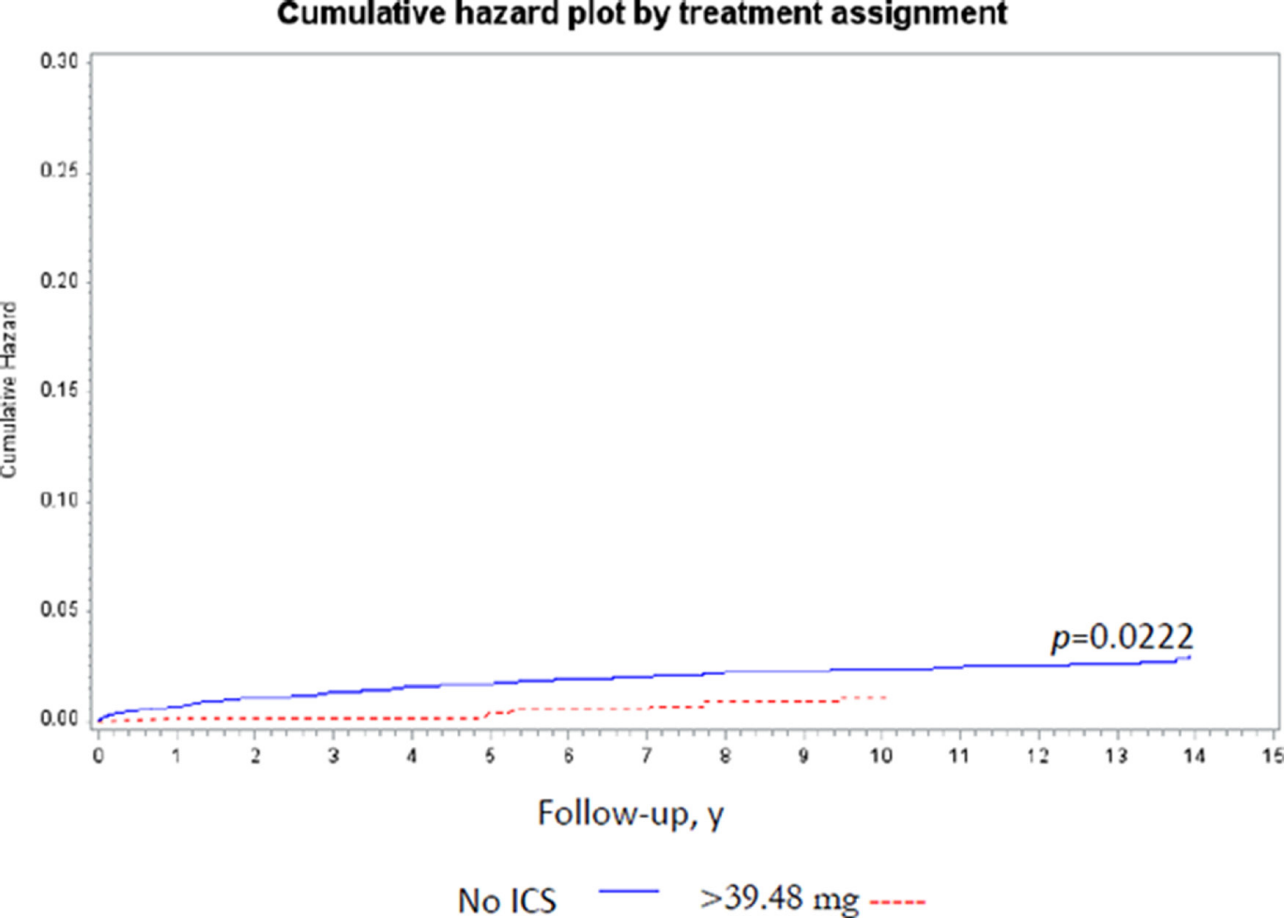
The cumulative lung cancer probability among ICS users (> 39.48 mg) and nonusers Note: The cumulative lung cancer probability significantly decreased among the ICs users compared with nonusers (*P* = 0.0222). Abbreviation: ICS: inhaled corticosteroids.

The time to lung cancer diagnosis after initial diagnosis of COPD was significantly different between ICS users and non-users (10.75 vs. 9.68 years, *P <* 0.001; Table 1). Lung cancer incidence with regard to variable ICS dose was determined adjusting for age, income, and comorbidities by Cox regression analyses (Table 2). The cumulative dose was calculated as duration of ICS use × dosage of ICS. A therapeutic dose of 39.48 mg ICS was used as the median value of the cumulative dose in each patient. The incidence rate of lung cancer per 100,000 person-years was 235.92 among ICS non-users and 158.67 among ICS users [ICS non-users, HR = 1; ICS users crude HR = 0.70 (95% confidence interval {CI}: 0.46–1.09)]. After adjusting for age, income, and comorbidities, ICS cumulative dose > 39.48 mg was associated with a lower risk of lung cancer [no ICS use, HR = 1.0; ICS use > 0 mg but ≤ 39.48 mg, HR = 0.95 (95% CI: 0.67–1.60); ICS use > 39.48 mg, HR = 0.45 (95% CI: 0.21–0.96)] by Model II Cox regression analyses (Table 2). The cumulative lung cancer probability among patients with cumulative ICS use > 39.48 mg compared with non-users was significant (*P* = 0.0222) (Figure 2). Model I Cox regression analyses showed that there was no significant difference between without ICS users and any ICS users adjusted by age, income, and comorbidities (Table 2). Model III Cox regression analyses showed the dose-duration-day (DDD) was not associated with the incidence of lung cancer adjusted by age, income, and comorbidities (Table 2).

**Table 2:** Multivariate analysis of lung cancer incidence in variable ICS dose adjusted for age, income, and comorbidities by cox regression mode

	No. of patients	No. of person-years	No. of patients with lung ca	Incident Rate (per 100,000 person-years)	Crude HR (95% CI)	Mode I Multivariate-Adjusted HR (95% CI)	Mode II Multivariate-Adjusted HR (95% CI)	Mode III Multivariate-Adjusted HR (95% CI)
Age								
≥ 40~< 50	2077	20435.73	15	73.40	1.00	1.00	1.00	1.00
≥ 50~< 60	2632	25993.40	26	100.03	1.37 (0.72~2.58)	1.52 (0.79~2.91)	1.52 (0.79~2.91)	1.52 (0.79~2.91)
≥ 60	8977	87389.62	264	302.10	4.11 (2.44~6.91)	4.36 (2.50~7.61)	4.34 (2.49~7.58)	4.34 (2.49~7.58)
Income (NTD)								
< 15840	7953	78218.91	194	248.02	1.00	1.00	1.00	1.00
≥ 15840~< 21900	1659	17303.76	59	340.97	1.42 (1.06~1.89)	1.20 (0.89~1.61)	1.19 (0.89~1.60)	1.19 (0.89~1.60)
≥ 21900~< 34800	3169	29896.16	47	157.21	0.62 (0.45~0.85)	0.72 (0.52~0.99)	0.72 (0.52~0.99)	0.72 (0.52~0.99)
≥ 34800	905	8399.93	5	59.52	0.23 (0.10~0.57)	0.49 (0.19~1.25)	0.49 (0.19~1.24)	0.49 (0.19~1.24)
ICS use								
No ICS use	12396	119953.57	283	235.92	1.00	1.00	1.00	
Any ICS use	1290	13865.20	22	158.67	0.70 (0.46~1.09)	0.71 (0.46~1.10)		
Cumulative dose (mg)								
> 0 mg~≤ 39.48 mg	668	6867.18	15	218.43	0.95 (0.67~1.60)		0.96 (0.57~1.61)	
> 39.48 mg	622	6998.02	7	100.03	0.45 (0.21~0.96)		0.46 (0.21~0.97)	
dose-duration-day (DDD)								
< 28 DDD	12951	125631.79	296	235.61	1.00			1.00
≥ 28 DDD~≤ 100 DDD	381	4154.89	5	120.34	0.54 (0.22~1.30)			0.53 (0.22~1.29)
> 100 DDD	354	4032.08	4	99.20	0.45 (0.17~1.21)			0.46 (0.17~1.23)
Medical disease								
Pulmonary tuberculosis								
Without	12703	123583.93	248	200.67	1.00	1.00	1.00	1.00
With	983	10234.83	57	556.92	2.87 (2.15~3.83)	2.65 (1.95~3.59)	2.65 (1.95~3.60)	2.66 (1.96~3.60)
Pneumonia								
Without	12525	121681.34	285	234.22	1.00	1.00	1.00	1.00
With	1161	12137.42	20	164.78	0.73 (0.46~1.14)	0.54 (0.34~0.86)	0.54 (0.34~0.86)	1.29 (0.92~1.80)
Bronchiectasis								
Without ICD494	12416	119980.56	259	215.87	1.00	1.00	1.00	1.00
With ICD494	1270	13838.20	46	332.41	1.63 (1.19~2.23)	1.28 (0.91~1.79)	1.29 (0.92~1.80)	1.29 (0.92~1.80)
Pulmonary fibrosis								
Without	13531	132174.84	298	225.46	1.00	1.00	1.00	1.00
With	155	1643.92	7	425.81	1.97 (0.93~4.16)	1.38 (0.64~2.98)	1.36 (0.63~2.94)	1.36 (0.63~2.92)
Hypertension								
Without	3460	31513.57	101	320.50	1.00	1.00	1.00	1.00
With	10226	102305.19	204	199.40	0.65 (0.51~0.82)	0.59 (0.46~0.75)	0.59 (0.46~0.75)	0.59 (0.46~0.75)
Diabetes mellitus								
Without	7624	71842.76	210	292.31	1.00	1.00	1.00	1.00
With	6062	61976.00	95	153.29	0.54 (0.43~0.69)	0.58 (0.45~0.74)	0.58 (0.45~0.74)	0.58 (0.45~0.74)

Abbreviations: NTD: New Taiwan Dollar.

### The relationship between risk of lung cancer and other variables

Being elderly was associated with a higher risk of lung cancer, and age ≥ 60 years led to an increased risk (HR = 4.34 [95% CI: 2.49–7.58]). Median and high income (21900–34800 and ≥ 34800 NTD per month, respectively) were associated with a lower risk of lung cancer compared to low income, but this was not significant. Pneumonia [multivariate-adjusted HR = 0.54 (95% CI: 0.34–0.96)], diabetes mellitus [multivariate-adjusted HR = 0.58 (95% CI: 0.45–0.74)], and hypertension [multivariate-adjusted HR = 0.59 (95% CI: 0.46–0.75)] were all significantly associated with a reduced risk of lung cancer. Pulmonary tuberculosis (TB) was significantly associated with an increased risk of lung cancer [HR = 2.65 (95% CI: 1.95–3.60)]. Bronchiectasis and pulmonary fibrosis were not associated with lung cancer risk.

## DISCUSSION

Our study demonstrated that ICS have a dose-dependent negative association with lung cancer risk, and that an ICS cumulative dose > 39.48 mg is significantly associated with a lower risk for lung cancer after adjusting for age, income, and comorbidities. The time to lung cancer diagnosis after initial diagnosis of COPD in ICS users was significantly longer than in ICS non-users; thus, ICS may have a protective effect against lung cancer. To account for these observations, the following mechanisms were considered. First, chronic inflammation has been found to play a role in cancer pathogenesis in a number of COPD cases. COPD is accompanied by an enhanced chronic inflammatory response in the airways and lungs [1], and ICS has the effect of reducing airway, lung, and systemic inflammation in COPD patients [17–19]. Previous studies have demonstrated that inhaled budesonide can decrease the number of neutrophils, as well as the levels of IL-6, TNF-α, and IL-8 in bronchoalveolar lavage samples in COPD patients [28, 29]. Smokers with COPD who have been prescribed inhaled fluticasone treatment showed decreased CD8/CD4 ratios and subendothelial mast cells in airway biopsies compared to patients using placebo [18]. ICS could also reduce C-reactive protein levels in COPD patients compared to other treatment regimens [30]. Inflammatory markers have also been found to increase during COPD exacerbations. Regular ICS treatments have reduced exacerbations in COPD patients [31], and withdrawal from ICS treatment has led to exacerbations in some patients [32]. The decrease in acute COPD exacerbations through ICS treatment reduces airway and systemic inflammation, thereby indirectly decreasing lung cancer incidence. Second, cyclooxygenase and prostaglandins have a major impact on lung tumor progression and tumor-associated inflammation. ICS may suppress proto-oncogenes in human smokers via modulating the COX-2 inflammatory pathway [15]. COX-2 is overexpressed in most non-small cell lung cancers (NSCLCs). COX-2 induced-prostaglandin E2 has been shown to be involved in anti-apoptosis, angiogenesis, tumor invasion, and inhibition of antitumor immunity. Therefore, COX-2 inhibitors have been applied to suppress the development and progression of lung cancer. Glucocorticoids are the most potent COX-2 inhibitors; they function by suppressing COX-2 expression through stimulating the glucocorticoid receptor (GR) [33–35] and reducing prostaglandin production through inhibition of cytosolic phospholipase A2 activity [36]. Third, ICS themselves may mediate a protective effect against lung cancer. *In vitro* studies have demonstrated that glucocorticoids have growth-inhibitory effects on NSCLC cell lines. Dexamethasone has been shown to exhibit growth-inhibitory effects on several GR-rich NSCLC cell lines [37]. Dexamethasone and budesonide have been shown to inhibit the proliferation of A549 cells, a GR-rich adenocarcinoma-derived human alveolar epithelial cell line [38, 39]. Budesonide has been shown to have chemopreventive efficacy on mouse lung tumorigenesis by modulating p53 and BclII protein expression [16]. Additionally, adrenalectomies have been demonstrated to enhance lung tumor formation in mouse models.

Raymakers et al. obtained several conflicting results about ICS effects on lung cancer in a systematic review. Their analysis of randomized controlled trials (RCTs) showed no statistically significant association between ICS use and lung cancer risk, whereas observational studies showed a protective effect of ICS use, particularly at higher doses and with more frequent use [27]. The primary objective of these RCTs was not to examine the protective effects of ICS on lung cancer, but the efficacy of ICS use in COPD. Low numbers of lung cancer events in the ICS treatment group and the controls were found in these RCTs during a short study period. It is possible that ICS do not protect against lung cancer in such a short follow-up time. Additionally, two observational studies showed a median time from COPD diagnosis to lung cancer of 2.2 and 1.4 years. Cancer latency periods tend to be quite long; thus, it must be considered that patients included in this study may have already had latent cancer. The time to lung cancer diagnosis after an initial diagnosis of COPD in our study population was significantly different between ICS users and non-users (10.75 vs. 9.68 years, *P <* 0.001).

The strengths of our study included its use of national population-based data that are highly representative of the general population. Our study had larger numbers (13,686 COPD patients) and a longer duration (the median follow-up period 9.78 years) than previous studies, which allowed for more data on COPD patients. When or whether a patient is prescribed ICS is decided by the physician according to COPD severity development, introducing another real world condition.

Additionally, our study also showed that age ≥ 60 years, pneumonia, diabetes mellitus, and hypertension were significantly associated with reduced risk of lung cancer, whereas pulmonary TB was associated with a higher risk of lung cancer.

Diabetic patients with COPD had a lower incidence of lung cancer than non-diabetic COPD patients. This requires further study to clarify whether this effect was due to diabetes itself or the antidiabetic medication. The literature shows that metformin and thiazolidinedione can reduce the incidence of lung cancer in patients with type 2 diabetes [41–44], but diabetes itself has no effect on the incidence of lung cancer [44]. This mechanism is due to the systemic effect of metformin in reducing the circulating level of insulin and insulin-like growth factor-1, which may associated with anticancer action [45]. Our study demonstrated that hypertension was associated with reduced lung cancer risk. β blockers [46–51] and captopril [51] are considered potential inhibitors of lung tumor growth and metastasis. β blockers may lower the risk of NSCLC development among smokers, and may be used to enhance the clinical outcome of standard cancer therapies. The antitumorigenic mechanisms of β blockers may be because of the inhibition or blockage of norepinephrine[46, 47] and nicotine effects [46], or inhibition of a beta-adrenergic receptor-mediated mitogenic pathway [49]. Treatment of LNM35 lung cancer cells with captopril was found to induce apoptosis [51].

This study showed that pulmonary TB in COPD patients increased the incidence of lung cancer, which is consistent with findings of previous reports [52–55]. Pulmonary TB may be associated with an increased risk of all major subtypes of lung cancer [52, 53, 55]; frequent x-ray exposure and chronic lung inflammation may be the mechanisms for lung cancer development [52–54]. Pneumonia was associated with reduced lung cancer risk in our study, which is consistent with previous studies [56]. However, there have been conflicting results [54]. The protective effect of pneumonia on lung cancer may reflect potential differences in immune responses, and further investigation is needed to confirm this.

Our results showed that treatment with ICS resulted in decreased lung cancer incidence. It also reduces the frequency of COPD exacerbations and improves quality of life. However, long-term high-dose ICS may have some deleterious effects, such as oral candidiasis, hoarse voice, skin bruises, pneumonia [57, 58], and pulmonary TB [59]. Triamcinolone Acetonide is associated with reduced bone density. Physicians should carefully weigh the potential risks, benefits, and costs associated with the use of ICS in individuals with COPD.

There were a number of important limitations associated with our study. First, we used ICD-9 diagnostic codes to enroll the patients; thus, we were unable to confirm COPD diagnosis by spirometry or differentiate severity by FEV1. Lung cancer may be related to COPD severity. However, many debates exist on the evaluation of COPD severity. The updated guidelines indicate that the evaluation of COPD severity needs a multidimensional approach. BODE (body mass index, airflow obstruction, dyspnea, and exercise capacity) score, comorbidities, and fibrinogen or CRP biomarkers are also considered important factors for COPD severity. Thus, multidimensional data collection is not easily done, even in a prospective study. In addition, ICS use is suggested in patients with severe and very severe COPD, according to GOLD guidelines. In theory, patients with severe and very severe COPD have a higher incidence of lung cancer than patients with mild and moderate COPD. However, our results show that patients with more severe COPD using ICS are less likely to have lung cancer, which further indicates that ICS reduced the incidence of lung cancer in COPD patients. Second, our study is a population-based cohort study. When or whether a patient starts to use ICS is decided by the physician. This eliminates interference by COPD stage and severity at initial enrollment. Third, the NHI database used to enroll the patients did not allow us to differentiate between squamous cell carcinoma and non-squamous cell carcinoma, despite the epidemiology and carcinogenic process being quite different between lung cancer subtypes. Fourth, we have concerns regarding medication adherence. Patients pay a part of all medical expenses under Taiwan's current health-care system. It is not logical that these patients would see a doctor regularly and not take prescribed medicines, yet still be willing to pay for long-term medical costs. Thus, we believe that, although we calculated ICS dose based on NHI records that cannot show the dose that the patients actually received, the difference should be very limited. Another important limitation of the study is the lack of our NHI data for important risk factors, e.g., detailed patient history of tobacco use, symptoms, diet conditions and occupational exposures.

In conclusion, this nationwide population-based cohort study with a 15-year duration and larger population adds positive evidences that ICS have a potential role in lung cancer prevention among female patients with COPD. However, a stronger prospective study design and data replications are necessary to validate our findings.

## MATERIALS AND METHODS

### Source of data

The NHI Program, which provides compulsory universal health insurance in Taiwan, was implemented March 1, 1995. Since then, 98% of the island's population receives all forms of health care services, including outpatient services, inpatient care, Chinese medicine, dental care, childbirth services, physical therapy, preventive health care, home care, and rehabilitation for chronic mental illness through this program. Most medical providers (93%) are under contract with the Bureau of NHI (BNHI), and those that are not under contract provide fewer health services. Consequently, more than 96% of Taiwan's population has insurance coverage through the NHI and has utilized health services at least once at contracted medical institutions. Based on the availability of the reimbursement claim records under a single payer system, a systematic sampling method was used by the National Health Research Institute (NHRI) of Taiwan to establish a randomly sampled and representative panel database of 1,000,000 patients. This database was established in 2000 for research purposes, and the NHRI has reported that there are no statistically significant differences in age, gender, and health care costs between the sample group and all enrollees [60].

Data regarding daily clinic visits and hospital admissions are available in an electronic format and can be obtained for each contracted medical institution. In order to be reimbursed, all medical institutions must submit their claims for billable medical services on a standardized electronic form, and this form includes data elements such as the date of admission and discharge, patient identification number, gender, birthday, and ICD-9-CM diagnostic code for each admission. Therefore, the information from the NHI database appears to be sufficiently complete, reliable, and accurate for use in epidemiological studies. This study was approved by the Institutional Review Board at the Chang-Gung Memorial Hospital (IRB #102-0364B). The requirement for informed consent was waived as the personal information used had already been de-identified in the NHI Research Database.

### Study design and participants

The worldwide average incidence of lung cancer in men is approximately 3-fold higher than that in women [61]. Taiwan has a particularly high male smoking prevalence, and but the prevalence in women is much lower. The ratio of male to female smoking rates is 10.9 to 1 among adults (46.8%/4.3%) in Taiwan [62], and about 15–20% of these female smokers may acquire COPD. Data about tobacco consumption and second-hand exposure could not be obtained from BNHI records. Therefore, to decrease the interference of sex and tobacco exposure in the incidence of lung cancer, we decided to select only female patients in this study. We identified an exposed study cohort from the database consisting of female patients newly diagnosed with COPD from 1996 to 2011. Patients were defined by the presence of two or more diagnostic codes for COPD (International Classification of Diseases, 9th revision, Clinical Modification (ICD-9-CM) 491, 492, 496) within 12 months in either inpatient or outpatient service claims submitted between 1996 and 2011 (*n* = 88,513). Patients were excluded if they were younger than 40 years, if lung cancer had been diagnosed prior to the diagnosis of COPD, or the patient had cases of asthma (ICD-9 CM code 493.X) before the index date. The index date for each participant was the date of first COPD diagnosis. A flowchart describing this process is shown in Figure 1. Potential confounders including age [63], income [64], and comorbidities such as pulmonary TB [52–55], pneumonia [54, 56], bronchiectasis [65], pulmonary fibrosis [66], hypertension [46–51], and diabetes [41–45] have been reported to be associated with lung cancer risk. Therefore, these factors were also included in this study.

### Exposure assessment

The ICS that were analyzed in this study included fluticasone propionate and budesonide. Information regarding exposure to ICS was extracted from the prescription database. Users of ICS were defined as those who received at least one prescription for an ICS between the COPD diagnosis date and index date. The date of prescription, daily dose, and number of days supplied were identified and cumulative doses (mg) were calculated. The use of ICS was approved in Taiwan in June 2001 and was placed on the listing of NHI drugs for reimbursement in February 2002. The period of an ICS prescription at 1 visit is at most 28 days in Taiwan. Therefore, ICS users were defined as patients who received an ICS prescription for > 28 days, and those who did not receive an ICS prescription were classified as nonusers of ICS. To evaluate the exposure effects of ICS, ICS dose-duration-day (DDD) according to WHO suggestion was cumulatively calculated as days of ICS prescription. (https://www.whocc.no/atc_ddd_index/)

### Lung cancer outcome and patient follow-up

The index date for each participant was the first date of COPD diagnosis. The study endpoint was established based on the first diagnosis of lung cancer (ICD-9 162.X) from outpatient claims or hospitalization records from 1997 to 2011. All of the study participants were followed from the index date to endpoint occurrence, withdrawal from the database, or the end of 2011, whichever came first.

### Statistical analysis

The person-years of follow-up for each case were calculated from the date of COPD diagnosis to the date of death or December 31, 2011. Incident rates were calculated by dividing the number of deaths from lung cancer by the number of person-years of follow-up. Model I was Cox regression analysis of relative risk of lung cancer incidence adjusted by age, income, and comorbidities and any ICS use. Model II was Cox regression analysis of relative risk of lung cancer incidence adjusted by age, income, and comorbidities and ICS cumulative dose. The participants were divided into three exposure categories: ICS non-users, ICS users of doses equal to or less than the median, and ICS users of doses greater than the median (median based on the distribution of use among all ICS cases). Hazard ratios and their 95% confidence intervals were calculated with non-user patients as a reference. To test the dosage effects of ICS, we further performed model III Cox regression analysis adjusted by tertiles of ICS DDD, by age, income, and comorbidities. ICS use was divided into tertiles by the DDD, and nonusers of ICS (< 28 DDD) were the reference point. Analyses were performed using the SAS statistical package (version 9.3; SAS Institute Inc., Cary, NC, USA). All statistical tests were two-sided and a *P-value* < 0.05 was considered statistically significant.

## Abbreviations

COPD: chronic obstructive pulmonary disease
ICS: inhaled corticosteroids
CI: confidence interval
HR: hazard ratio
IRB: institutional review board
NHI: National Health Insurance
NHRI: National Health Research Institute
ICD-9-CM: International Classification of Diseases, Ninth Revision- Clinical Modification
BNHI: Bureau of National Health Insurance
COX-2: cyclooxygenase-2
CRP: C-reactive protein
GOLD: Global Initiative for Chronic Obstructive Pulmonary Disease
NSCLC: non-small cell lung cancer
TB: tuberculosis
GR: glucocorticoid receptor
NTD: New Taiwan Dollar
BODE: body mass index, airflow obstruction, dyspnea, and exercise capacity
FEV1: a forced expiratory volume in the first second
DDD: dose-duration-day

## References

[1] Global Strategy for the Diagnosis, Management and Prevention of COPD, Global Initiative for Chronic Obstructive Lung Disease (GOLD) (2016). http://goldcopd.org/.

[2] Anthonisen NR, Connett JE, Enright PL, Manfreda J, Lung Health Study Research Group (2002). Hospitalizations and mortality in the Lung Health Study. Am J Respir Crit Care Med.

[3] Torre LA, Bray F, Siegel RL, Ferlay J, Lortet-Tieulent J, Jemal A (2015). Global cancer statistics, 2012. CA Cancer J Clin.

[4] Purdue MP, Gold L, Järvholm B, Alavanja MCR, Ward MH, Vermeulen R (2007). Impaired lung function and lung cancer incidence in a cohort of Swedish construction workers. Thorax.

[5] Mannino DM, Aguayo SM, Petty TL, Redd SC (2003). Low lung function and incident lung cancer in the United States: data from the first National Health and Nutrition Examination Survey follow-up. Arch Intern Med.

[6] Turner MC, Chen Y, Krewski D, Calle EE, Thun MJ (2007). Chronic obstructive pulmonary disease is associated with lung cancer mortality in a prospective study of never smokers. Am J Respir Crit Care Med.

[7] Young RP, Hopkins RJ, Christmas T, Black PN, Metcalf P, Gamble GD (2009). COPD prevalence is increased in lung cancer, independent of age, sex and smoking history. Eur Respir J.

[8] Tan WC, Sin DD, Bourbeau J, Hernandez P, Chapman KR, Cowie R, FitzGerald JM, Marciniuk DD, Maltais F, Buist AS, Road J, Hogg JC, Kirby M, CanCOLD Collaborative Research Group (2015). Characteristics of COPD in never-smokers and ever-smokers in the general population: results from the CanCOLD study. Thorax.

[9] Spencer S, Calverley PM, Burge PS, Jones PW (2004). Impact of preventing exacerbations on deterioration of health status in COPD. Eur Respir J.

[10] Shacter E, Weitzman SA (2002). Chronic inflammation and cancer. Oncology.

[11] Schetter AJ, Heegaard NH, Harris CC (2010). Inflammation and cancer: interweaving microRNA, free radical, cytokine and p53 pathways. Carcinogenesis.

[12] Durham AL, Adcock IM (2015). The relationship between COPD and lung cancer. Lung Cancer.

[13] Greenberg AK, Hu J, Basu S, Hay J, Reibman J, Yie TA, Tchou-Wong KM, Rom WN, Lee TC (2002). Glucocorticoids inhibit lung cancer cell growth through both the extracellular signal-related kinase pathway and cell cycle regulators. Am J Resp Cell Mol Biol.

[14] Yao R, Wang Y, Lemon WJ, Lubet RA, You M (2004). Budesonide exerts its chemopreventive efficacy during mouse lung tumorigenesis by modulating gene expression. Oncogene.

[15] Brown JR, DuBois RN (2004). Cyclooxygenase as a target in lung cancer. Clin Cancer Res.

[16] Lam S, leRiche JC, McWilliams A, Macaulay C, Dyachkova Y, Szabo E, Mayo J, Schellenberg R, Coldman A, Hawk E, Gazdar A (2004). A randomized phase IIb trial of Pulmicort Turbuhaler (budesonide) in people with dysplasia of the bronchial epithelium. Clin Cancer Res.

[17] Gizycki MJ, Hattotuwa KL, Barnes N, Jeffery PK (2002). Effects of fluticasone propionate on inflammatory cells in COPD: an ultrastructural examination of endobronchial biopsy tissue. Thorax.

[18] Hattotuwa KL, Gizycki MJ, Ansari TW, Jeffery PK, Barnes NC (2002). The effects of inhaled fluticasone on airway inflammation in chronic obstructive pulmonary disease: a double-blind, placebo-controlled biopsy study. Am J Respir Crit Care Med.

[19] Löfdahl CG, Postma DS, Pride NB, Boe J, Thorén A (2007). Possible protection by inhaled budesonide against ischaemic cardiac events in mild COPD. Eur Respir J.

[20] Parimon T, Chien JW, Bryson CL, McDonell MB, Udris EM, Au DH (2007). Inhaled corticosteroids and risk of lung cancer among patients with chronic obstructive pulmonary disease. Am J Respir Crit Care Med.

[21] Kiri VA, Fabbri LM, Davis KJ, Soriano JB (2009). Inhaled corticosteroids and risk of lung cancer among COPD patients who quit smoking. Respir Med.

[22] Pauwels RA, Löfdahl C-G, Laitinen LA, Schouten JP, Postma DS, Pride NB, Ohlsson SV (1999). European Respiratory Society Study on Chronic Obstructive Pulmonary Disease. Long-term treatment with inhaled budesonide in persons with mild chronic obstructive pulmonary disease who continue smoking. N Engl J Med.

[23] Tashkin DP, Rennard SI, Martin P, Ramachandran S, Martin UJ, Silkoff PE, Goldman M (2008). Efficacy and safety of budesonide and formoterol in one pressurized metered-dose inhaler in patients with moderate to very severe chronic obstructive pulmonary disease. Drugs.

[24] Burge PS, Calverley PM, Jones PW, Spencer S, Anderson JA, Maslen TK (2000). Randomised, double blind, placebo controlled study of fluticasone propionate in patients with moderate to severe chronic obstructive pulmonary disease: the ISOLDE trial. Br Med J.

[25] Kok VC, Horng JT, Huang HK, Chao TM, Hong YF (2015). Regular inhaled corticosteroids in adult-onset asthma and the risk for future cancer: a population-based cohort study with proper person-time analysis. Ther Clin Risk Manag.

[26] Lee CH, Hyun MK, Jang EJ, Lee NR, Kim K, Yim JJ (2013). Inhaled corticosteroid use and risks of lung cancer and laryngeal cancer. Respir Med.

[27] Raymakers AJ, McCormick N, Marra CA, Fitzgerald JM, Sin D, Lynd LD (2017). Do inhaled corticosteroids protect against lung cancer in patients with COPD? A systematic review. Respirology.

[28] Ozol D, Aysan T, Solak ZA, Mogulkoc N, Veral A, Sebik F (2005). The effect of inhaled corticosteroids on bronchoalveolar lavage cells and IL-8 levels in stable COPD patients. Respir Med.

[29] Ko FW, Leung TF, Wong GW, Ngai J, To KW, Ng S, Hui DS (2009). Measurement of tumor necrosis factor-alpha, leukotriene B4, and interleukin 8 in the exhaled breath condensate in patients with acute exacerbations of chronic obstructive pulmonary disease. Int J Chron Obstruct Pulmon Dis.

[30] Sin DD, Lacy P, York E, Man SF (2004). Effects of fluticasone on systemic markers of inflammation in chronic obstructive pulmonary disease. Am J Respir Crit Care Med.

[31] Calverley PM, Anderson JA, Celli B, Ferguson GT, Jenkins C, Jones PW, Yates JC, Vestbo J, TORCH investigators (2007). Salmeterol and fluticasone propionate and survival in chronic obstructive pulmonary disease. N Engl J Med.

[32] van der Valk P, Monninkhof E, van der Palen J, Zielhuis G, van Herwaarden C (2002). Effect of discontinuation of inhaled corticosteroids in patients with chronic obstructive pulmonary disease: the COPE study. Am J Respir Crit Care Med.

[33] Chang J, Xue M, Yang S, Yao B, Zhang B, Chen X, Pozzi A, Zhang MZ (2015). Inhibition of 11β-Hydroxysteroid Dehydrogenase Type II Suppresses Lung Carcinogenesis by Blocking Tumor COX-2 Expression as Well as the ERK and mTOR Signaling Pathways. PLoS One.

[34] Clark AR, Lasa M (2003). Cross talk between glucocorticoids and mitogen-activated proteinkinase signaling pathways. Curr Opin Pharmacol.

[35] Newton R (2000). Molecular mechanisms of glucocorticoid action: what is important?. Thorax.

[36] Stichtenoth DO, Thoren S, Bian H, Peters-Golden M, Jakobsson PJ, Crofford LJ (2001). Microsomal prostaglandin E synthaseis regulated by proinflammatory cytokines and glucocorticoids in primary rheumatoid synovial cells. Immunol.

[37] Hofmann J, Kaiser U, Maasberg M, Havemann K (1995). Glucocorticoid receptors and growth inhibitory effects of dexamethasone in human lung cancer cell lines. Eur J Cancer.

[38] Liang H, Kowalczyk P, Junco JJ, Klug-De Santiago HL, Malik G, Wei SJ, Slaga TJ (2014). Differential effects on lung cancer cell proliferation by agonists of glucocorticoid and PPARalpha receptors. Mol Carcinog.

[39] Gundisch S, Boeckeler E, Behrends U, Amtmann E, Ehrhardt H, Jeremias I (2012). Glucocorticoids augment survival and proliferation of tumor cells. Anticancer Res.

[40] Droms KA, Fernandez CA, Thaete LG, Malkinson AM (1988). Effects of drenalectomy and corticosterone administration on mouse lung tumor susceptibility and histogenesis. J Natl Cancer Inst.

[41] Tsai MJ, Yang CJ, Kung YT, Sheu CC, Shen YT, Chang PY, Huang MS, Chiu HC (2014). Metformin decreases lung cancer risk in diabetic patients in a dose-dependent manner. Lung Cancer.

[42] Lin HC, Hsu YT, Kachingwe BH, Hsu CY, Uang YS, Wang LH (2014). Dose effect of thiazolidinedione on cancer risk in type 2 diabetes mellitus patients: a six-year population-based cohort study. J Clin Pharm Ther.

[43] Mazzone PJ, Rai H, Beukemann M, Xu M, Jain A, Sasidhar M The effect of metformin and thiazolidinedione use on lung cancer in diabetics. BMC Cancer.

[44] Lai SW, Liao KF, Chen PC, Tsai PY, Hsieh DP, Chen CC (2012). Antidiabetes drugs correlate with decreased risk of lung cancer: a population-based observation in Taiwan. Clin Lung Cancer.

[45] Memmott RM, Mercado JR, Maier CR, Kawabata S, Fox SD, Dennis PA (2010). Metformin prevents tobacco carcinogen—induced lung tumorigenesis. Cancer Prev Res. (Phila).

[46] Al-Wadei HA, Al-Wadei MH, Schuller HM (2012). Cooperative regulation of non-small cell lung carcinoma by nicotinic and beta-adrenergic receptors: a novel target for intervention. PLoS One.

[47] Fitzgerald PJ (2009). Is norepinephrine an etiological factor in some types of cancer?. Int J Cancer.

[48] Fitzgerald PJ (2010). Testing whether drugs that weaken norepinephrine signaling prevent or treat various types of cancer. Clin Epidemiol.

[49] Park PG, Merryman J, Orloff M, Schuller HM (1995). Beta-adrenergic mitogenic signal transduction in peripheral lung adenocarcinoma: implications for individuals with preexisting chronic lung disease. Cancer Res.

[50] Lin CS, Lin WS, Lin CL, Kao CH (2015). Carvedilol use is associated with reduced cancer risk: A nationwide population-based cohort study. Int J Cardiol.

[51] Attoub S, Gaben AM, Al-Salam S, Al Sultan MA, John A, Nicholls MG, Mester J, Petroianu G (2008). Captopril as a potential inhibitor of lung tumor growth and metastasis. Ann N Y Acad Sci.

[52] Yu YH, Liao CC, Hsu WH, Chen HJ, Liao WC, Muo CH, Sung FC, Chen CY (2011). Increased lung cancer risk among patients with pulmonary tuberculosis: a population cohort study. J Thorac Oncol.

[53] Cicenas S, Vencevicius V (2007). Lung cancer in patients with tuberculosis. World J Surg Oncol.

[54] Wu MF, Jian ZH, Huang JY, Jan CF, Nfor ON, Jhang KM, Ku WY, Ho CC, Lung CC, Pan HH, Wu MC, Liaw YP (2016). Post-inhaled corticosteroid pulmonary tuberculosis and pneumonia increases lung cancer in patients with COPD. BMC Cancer.

[55] Jian ZH, Huang JY, Lin FC, Nfor ON, Jhang KM, Ku WY, Ho CC, Lung CC, Pan HH, Wu MC, Wu MF, Liaw YP Post-Inhaled Corticosteroid Pulmonary Tuberculosis Increases Lung Cancer in Patients with Asthma. PLoS One.

[56] Koshiol J, Rotunno M, Consonni D, Pesatori AC, De Matteis S, Goldstein AM, Chaturvedi AK, Wacholder S, Landi MT, Lubin JH, Caporaso NE (2010). Lower Risk of Lung Cancer after Multiple Pneumonia Diagnoses. Cancer Epidemiol Biomarkers Prev.

[57] Kew KM, Seniukovich A Inhaled steroids and risk of pneumonia for chronic obstructive pulmonary disease. Cochrane Database Syst Rev.

[58] Singh S, Amin AV, Loke YK (2009). Long-term use of inhaled corticosteroids and the risk of pneumonia in chronic obstructive pulmonary disease: a meta-analysis. Arch Intern Med.

[59] Lee CH, Kim K, Hyun MK, Jang EJ, Lee NR, Yim JJ (2013). Use of inhaled corticosteroids and the risk of tuberculosis. Thorax.

[60] Kuo HC, Chang WC, Yang KD, Yu HR, Wang CL, Ho SC, Yang CY (2013). Kawasaki disease and subsequent risk of allergic diseases: a population-based matched cohort study. BMC Pediatr.

[61] Gender in lung cancer and smoking research http://search.who.int/search?q=Gender+in+lung+cancer+and+smoking+research&ie=utf8&site=who&client=_en_r&proxystylesheet=_en_r&output=xml_no_dtd&oe=utf8&getfields=doctype.

[62] Wen CP, Levy DT, Cheng TY, Hsu CC, Tsai SP (2001). Smoking behaviour in. Taiwan.

[63] White MC, Holman DM, Boehm JE, Peipins LA, Grossman M, Henley SJ (2014). Age and cancer risk: a potentially modifiable relationship. Am J Prev Med.

[64] Mitra D, Shaw A, Tjepkema M, Peters P (2015). Social determinants of lung cancer incidence in Canada: A 13-year prospective study. Health rep.

[65] Chung WS, Lin CL, Hsu WH, Kao CH (2016). Increased risk of lung cancer among patients with bronchiectasis: a nationwide cohort study. QJM.

[66] Kumar P, Goldstraw P, Yamada K, Nicholson AG, Wells AU, Hansell DM, Dubois RM, Ladas G (2003). Pulmonary fibrosis and lung cancer: risk and benefit analysis of pulmonary resection. J Thorac Cardiovasc Surg.

